# Role of Macrophages and Microglia in Zebrafish Regeneration

**DOI:** 10.3390/ijms21134768

**Published:** 2020-07-05

**Authors:** Susanna R. Var, Christine A. Byrd-Jacobs

**Affiliations:** Department of Biological Sciences, Western Michigan University, Kalamazoo, MI 49008-5410, USA; susanna.r.var@wmich.edu

**Keywords:** immunology, neuroregeneration, fish, immune cells, zebrafish, microglia, macrophage

## Abstract

Currently, there is no treatment for recovery of human nerve function after damage to the central nervous system (CNS), and there are limited regenerative capabilities in the peripheral nervous system. Since fish are known for their regenerative abilities, understanding how these species modulate inflammatory processes following injury has potential translational importance for recovery from damage and disease. Many diseases and injuries involve the activation of innate immune cells to clear damaged cells. The resident immune cells of the CNS are microglia, the primary cells that respond to infection and injury, and their peripheral counterparts, macrophages. These cells serve as key modulators of development and plasticity and have been shown to be important in the repair and regeneration of structure and function after injury. Zebrafish are an emerging model for studying macrophages in regeneration after injury and microglia in neurodegenerative disorders such as Parkinson’s disease and Alzheimer’s disease. These fish possess a high degree of neuroanatomical, neurochemical, and emotional/social behavioral resemblance with humans, serving as an ideal simulator for many pathologies. This review explores literature on macrophage and microglial involvement in facilitating regeneration. Understanding innate immune cell behavior following damage may help to develop novel methods for treating toxic and chronic inflammatory processes that are seen in trauma and disease.

## 1. Introduction

The immune system is generally divided into the nonspecific innate and the specific adaptive immune system. The innate immune system consists of the nonspecific reaction by immune cells that attack foreign antigens. The adaptive immune system is antigen specific and involves immune cells that attack pathogens in an initial response followed by subsequent reactions involving the same antigen. This review will focus on the innate immune system, particularly the roles of macrophages and microglia in tissue and neural regeneration in fish and its potential translational impact in regeneration studies. 

The innate immune system is a nonspecific, evolutionarily conserved defense strategy, responsible for initiating reactions to eliminate potentially damaging and infectious threats in vertebrates, including fish. Innate immune mechanisms include the recruitment of immune cells such as macrophages and microglia to facilitate the removal of tissues and toxic molecules from the circulation and parenchyma as a result of damage from injury, aging, or necrosis [1,2]. Additional consequences of this response can be harmful or helpful, contributing to further damage or to recovery. This review will focus primarily on the contribution of macrophages and microglia in regeneration in the zebrafish.

## 2. Overview of Physiological Functions of Macrophages and Microglia

Macrophages were discovered in 1882 by Russian zoologist and immunologist Ilya Metchnikoff and are considered to be an evolutionarily conserved defense mechanism in innate immunity [3,4]. In 1968, van Furth proposed a new classification of macrophages, monocytes, and their precursor cells as part of the mononuclear phagocyte system—today comprising monocytes, macrophages, dendritic cells, and bone marrow-resident progenitors—that originated from circulating blood monocytes. This view prevailed for subsequent decades [5,6,7,8,9]. There are many distinct macrophage subsets with different functional abilities; however, macrophages primarily ingest and destroy pathogens and remove dead cells and debris to maintain healthy tissue [10]. Macrophages play a role in the maintenance of tissue architecture during development, restoration after injury, and resolution of inflammation, bridging the relationship between the innate and adaptive immune systems through surveillance activities, phagocytosis, cytokine production, immune cell recruitment, and antigen presentation [1,11]. More recently, macrophage origin is considered to be established during embryonic development—independent of blood monocyte contribution during adulthood—and differentiate in the yolk sac of mammals, birds, and zebrafish, implying that macrophages serve a significant role in the early embryo [12,13,14,15,16].

The innate immune system relies on a species’ adaptation to specific environmental conditions through a variety of germline-encoded pattern-recognition receptors (PRRs) [17,18,19,20]. These molecular patterns include Toll-like receptors (TLRs), foreign or pathogen-associated molecular patterns (PAMPs), or molecular patterns that result from the damage of host tissue due to injury and infection, called damaged associated molecular patterns (DAMPs). TLRs are receptors expressed in innate immune cells, among other cells, that are involved in defense against invading microorganisms, mainly by recognizing PAMPs such as bacterial lipopolysaccharides and endotoxins and viral RNA. DAMPS are molecules released from damaged cells that promote inflammation; these include high-mobility group box 1 (HMGB1), S100 proteins, and heat-shock proteins. PRRs are highly conserved in mammals and fish [21,22,23] and have the capacity to change gene expression in macrophage metabolism, influencing subsequent inflammatory activities [24,25]. PRRs, PAMPs, DAMPs, and other chemically distinct cues from neighboring cells allow macrophages to respond in a variety of ways, ranging from supporting cell survival through the production of ligands or facilitating cell death [26,27,28,29]. Macrophages also display a tissue-specific heterogeneity with distinct patterns of gene expression and cell-surface receptor proteins [11]. Tissue-specific heterogeneity refers to the developmentally specific existence and operation of macrophages within a specific tissue and adapting to that particular environment [30,31,32]. 

Macrophages that operate residentially in the central nervous system (CNS) are termed microglia. Microglia were discovered in 1919 by Spanish neuroscientist Pío del Río Hortega, and the following 100 years of microglial research yielded the idea that these cells were the innate immune cells of the CNS. Today, microglia are understood to be macrophage-like innate immune cells with memory-like functions that exist in CNS tissue. Physiologically, microglia show a high degree of plasticity, and their ability to transform rapidly from a resting sensorial cell to an active phagocytic state is well described [33,34,35]. 

Increasing evidence has demonstrated that microglia also act as key modulators in neuronal development and plasticity, supporting and shaping brain tissue. Through their interaction with many other cells in the CNS, including neurons, astrocytes, and oligodendrocytes, this modulating feature differentiates microglia from other phagocytes that function primarily in innate immunity [36,37,38]. Major advances in sequencing technologies has revealed microglia also to be determinants of diseases rather than bystanders of CNS pathologies, such as Alzheimer’s disease, Parkinson’s disease, schizophrenia, autism and multiple sclerosis [39,40,41,42,43]. As a result, microglia have emerged as a novel and promising treatment target for many brain injuries, diseases, and disorders. 

Microglia are considered as macrophages that are part of the mononuclear phagocyte system. Their persistence from the embryonic brain throughout adulthood inevitably allows this cell type to encounter very distinct physiological states and environmental changes throughout the course of their existence. Under particular disease states or injury conditions, other leukocytes, such as neutrophils, lymphocytes, and monocytes, can travel to the CNS, migrating through blood vessels and cranial nerves to provide additional clearing of debris [14,44,45]. Like other embryonic tissue-specific macrophages, microglia are believed to originate from the yolk sack and are established at about the same time as neurons in the early prenatal period absent of other glial cells [14,36,46,47,48]. They are key players involved in the establishment of the neuronal architecture of the CNS, controlling neuronal fates and numbers, and engulfing dead and dying cells as well as excess synapses in the developing brain parenchyma [49,50,51]. Such developmental maintenance processes continue into adulthood, where microglia also shape neuronal circuits by clearing excess neurons and stimulate neurogenesis by assisting in the maturation, proliferation, and survival of neural progenitor cells and neurons [36,52]. Rather than simply play a role in the removal of nonfunctional synapses, microglia have also been shown to actively remodel synaptic circuits by partaking in synaptic pruning through ATP and the expression of the purinergic receptor P2Y12 [38,53,54,55].

Unlike peripheral macrophages, microglial cells are believed to be the only adult macrophage population with early erythroid myeloid progenitor origin (eEMP) [56,57]. eEMPs also include adult CNS macrophages that are protected by the blood-brain barrier. The persistence of these eEMP-derived microglia from the embryonic brain throughout adulthood requires specific cellular maintenance strategies, such as the dependence on constant stimulation of their CsF1 receptors: Csfr, MCSF-R, and CD115 [58,59]. Additionally, microglia have a remarkable potential for self-renewal by undergoing significant turnover through a balance of apoptosis and proliferation through IL-1 receptor signaling [60,61,62].

The interaction between the nervous and immune systems affects not only development across lifespan but also recovery after injury and disease [63,64]. It is recognized that microglial transformation in response to damage and disease in the CNS occurs as a continuum. Through changes involving gene expression, morphology, migration, metabolism, proliferation, and death, microglia transform in response to CNS pathology. Monocytes, macrophages, and microglia polarize to the pro-inflammatory type (M1) or protective anti-inflammatory type (M2) based on environmental conditions [65,66]. M1-like responses involve the upregulation of pro-inflammatory cytokines, including inducible nitric oxide synthase, interleukin (IL)-1β, IL-6, and tumor necrosis factor (TNF)-α. M2-like responses involve the upregulation of transforming growth factor (TGF)-β, cytokines IL-4, IL-10, and CD206 and CD163 [67,68,69]. Brain disease and injury involve the activation of resident and peripheral immune cells to clear damaged and dying neurons [43], and microglial processes will converge toward the site of CNS damage within minutes after acute injury. Within hours to days, microglia extend and retract their processes and transform into an activated, amoeboid form, where they readily engage in releasing inflammatory mediators [33,70,71,72].

Following tissue damage, the timely recruitment and migration of innate immune cells is often followed by phagocytosis. This phase of the injury response enables control of inflammation and sets the stage for tissue repair and functional recovery of the brain. The phagocytic behavior of innate immune cells is potentially important in the establishment of new axonal connections, as degenerating connections must first be removed for this event to occur [73,74]. Recent studies found incomplete restoration of function in mice following inadequate recruitment of monocyte-derived macrophages to the brain after injury [75]. It is unclear which immune cells are involved in the brain recovery process after injury, particularly in the olfactory system, an area of continual development with persistent neurogenesis and continual axon replacement. Understanding the control of innate immune cell behavior could potentially benefit functional recovery after neuronal damage seen in various neurodegenerative diseases, such as Alzheimer’s disease, Parkinson’s disease, stroke, or trauma, by providing a method for modifying the chronic neuroinflammatory processes [39,40,42,43].

Improving recovery of neuronal function after brain injuries and neurodegenerative diseases is a current and ongoing problem. Therapeutic interventions face common challenges such as generating neurons after loss of function and limiting the secondary tissue damage induced by long-term inflammation from accumulated immune cells including microglia at the injury site. While mammals, largely represented by the mouse model, prove to be the tractable animal model for use in studies that potentially could translate to clinical uses, mammals lack the regenerative capacity that is found in other vertebrate models such as the zebrafish. Contrary to mammalian models, *Danio rerio*, or zebrafish, have an extensive regenerative capacity in response to injuries, with specific mechanisms that promote the loss of tissue architecture and restoration of functionality in the CNS. Additionally, if the microglial response and resolution pertains to the capacity of the system to regenerate, then there is a great deal to learn from these animal models. Fortuitously, major organs and tissues of zebrafish share molecular, anatomical, and physiological similarities with their mammalian counterparts and possess over 70% of genes shared with humans [76,77,78].

A key difference between mouse models and the zebrafish model is that zebrafish possess many constitutively active neurogenic niches in the adult CNS [79,80,81], particularly in the olfactory system with its diverse plasticity mechanisms [82,83,84]. The regenerative nature of the zebrafish is useful for examining the potential of multi-organ regeneration after damage from injury or disease, including the brain, spinal cord, retina, fin, and heart [85]. 

## 3. Macrophages and Microglia in Tissue Regeneration in Fish

Macrophages are functionally diverse and highly plastic in homeostatic conditions and following injury or disease. For this reason, macrophages are heavily studied in fish regeneration studies (Table 1). Following damage, the shift from the tissue repair phase to a regenerative phase requires macrophages to play a role in both repair and tissue regeneration [86,87]. The expression of pro-inflammatory macrophages via TNF-α after an initial injury in zebrafish is similar to mammals, apparent when pro-inflammatory M1-like macrophages expressing TNFβ, IL-β, IL-6 are attracted to the site of injury to clean up foreign material and debris [88,89,90]. The downregulation of TNF-α expression is a hallmark for wound resolution and regeneration in zebrafish.

Macrophages are an essential player in heart regeneration after injury. Several species possess the capability of regenerating the injured heart with or without scar formation [92,98,99], but the zebrafish is capable of complete heart regeneration without stable scar formation, in contrast to humans which are incapable of regenerating the injured heart. Interestingly, a depletion of macrophages leads to a halt in regenerative processes of the heart [91,93,95,97], supporting the vital role of macrophages in recovery from damage. Understanding the mechanisms involved in zebrafish heart regeneration is an emerging area of research.

In the zebrafish, with its high regenerative capacity, macrophages are also necessary for the regeneration of fins [87,97,100]. Caudal fin amputation results in an accumulation of pro-inflammatory macrophages regulated by TNF-α transiently expressed by polarized macrophages during early phases of regeneration. This prepares the tissue for efficient regeneration and precedes the presence of M2-polarized macrophages that express TGFβ-1, chemokine receptor (CCR) 2, and cxcr4b, often thought to be preferentially associated with tissue repair [88,89]. Macrophages may also regulate aspects of appendage regeneration through Wnt/β-catenin signaling. Wnt signaling directly affects macrophage proliferation and cytokine release, modulating appendage inflammation and regeneration as well as regulating proliferation after injury in other structures, such as the optic tectum of adult zebrafish [87,111]. These findings suggest that Wnt signaling may play a significant role in the regeneration of different parts of the CNS, including the hypothalamus, retina, and spinal cord [119,126,127,128].

Like their peripheral counterparts, microglia serve as an important cellular population involved in homeostatic maintenance of the fish brain. These cells display varying degrees of proliferation and, under normal conditions, exist in their ramified, resting morphology, as they do in the mammalian brain. Likewise, zebrafish microglia also transform into an amoeboid, activated morphology under pathological conditions [72]. Their task as CNS guardians plays an important role in synaptic degeneration [129,130,131,132], regulation of neuronal components [80,122,133,134,135,136], clearance of apoptotic cells [137,138,139], and CNS angiogenesis and vascular maintenance [140,141,142].

Studies of microglial behavior in fish are becoming increasingly popular (Table 1), particularly those focused on the microglial response to injury via phagocytosis [50,52,143] and neuronal degeneration [144,145,146]. The phagocytic behavior of immune cells is potentially important in the establishment of new connections in the brain, as degenerating connections first must be removed before new connections can form [147]. For example, injury in the zebrafish is followed quickly by a microglial inflammatory response, characterized by morphological modification and leukocyte accumulation at the injury site [112,120,122,148]. There is also an increase in proliferation of ependymal glial cells that produces intermediate neural progenitors [85,149] that aid in replacing damaged neurons. Microglial cells abandon the injury site once sigma-1 receptors switch off [150], leading to a quickly resolved inflammatory response in the zebrafish telencephalon. Sigma-1 receptors are intracellular proteins in neurons and glia known to play a role in neurodegeneration. Studies have begun to explore the recruitment of both resident microglia and peripheral macrophages to the brain injury and their different roles during the response to neuronal cell death [43,151,152], addressing questions related to the individual contributions of two separate populations. 

Microglia have been shown to be highly dependent on macrophage colony-stimulating factor receptor (CSF1R) [153,154,155], which is broadly expressed by monocytes. The ligands macrophage colony-stimulating factor (mCSF) and Interleukine-34 (IL-34) bind to CSF1R, playing a role in the proliferation of innate immune cells, notably the monocyte and macrophage population. In the brain, CSF1R acts on the proliferation, survival, and phagocytosis of microglia [156]. This signaling pathway is involved in clearing myelin debris and toxic byproducts from the cerebral environment, serving a neuroprotective role. Some studies have found that inhibition of the CSF1R pathway in microglia does not impair microglial proliferation, indicating that mCSF receptor may not be necessary for proliferation in this particular model using a pathologic context of cuprizone-induced demyelination CSF1R signaling pathway. Additionally, CSF1R knock-out mice exhibited significant microgliosis, with no sign of impairment in response to injury [154]. Further exploration of microglial repopulation kinetics shows that microglial repopulation is proportional to the extent of microglial depletion, with the brain only having the capacity for a single complete repopulation event in mice [157]. 

Other studies in mice used a specific tyrosine kinase inhibitor to show the critical role of CSF1R in microglia and that repopulation is derived solely from residual microglia after acute depletion. However, it has been pointed out that manipulation of a molecule may affect various populations of cells and not specifically microglia [158]. Suppression of CSF1R also leads to infiltration of circulating CX3CR1-positive cells after surgery, where most proliferating microglia appeared to be resident Iba1 cells in mice [159]. The method of recruitment of bone marrow-derived cells is not well understood, and deletion of one receptor may lead to the overexpression in another to compensate for the deletion in microglia. Additionally, studies have shown that the brain can repopulate with myeloid cells despite the presence of surviving microglia in the brain after depletion, finding that the brain prefers to only have a single repopulation event [157]. 

Gene expression analyses have also shown repopulation by microglia in an activated state with an IFNγ-activated gene signature and upregulation of many pro-inflammatory cytokine and chemokines [160]. The ability of the microglia to repopulate the brain at specific timepoints has potential for therapeutic applications. For example, after traumatic brain injury, there is chronic activation of microglia that hinder functional recovery [161,162,163,164,165]; therefore, removal of reactive microglia followed by repopulation with fresh microglial cells offers a potential strategy to resolve neuroinflammation and encourage recovery [166]. 

## 4. Translational Studies in Fish and Mammalian Models

Study of microglial involvement in recovery and regeneration in fish has a potential translational impact, much like the mammalian model. For example, the anatomical components of the zebrafish olfactory system are comparable with other vertebrates, and the olfactory bulb is replete with a resident population of microglia [72,167]. The olfactory sensory neurons (OSNs) of the olfactory organ are continually replaced throughout life, under natural and injury conditions. This constitutive turnover requires perpetual removal of OSN axons in the central target olfactory bulb. Manipulations of the olfactory organ in mammals results in microglial activation in the olfactory bulb [168]; however, the role of these innate immune cells in the remarkable capacity of the olfactory system to recover both morphologically and functionally in zebrafish is unclear. The zebrafish olfactory system can serve as a model for neuroplasticity and disease, since damage to the olfactory system can alter morphological structure of OSNs and impair olfactory function. The effects of injury can be studied by examining olfactory organ morphology, neuronal activity and structure, as well as inflammatory and cellular alterations. Repetitive peripheral damage to the olfactory organ showed astrogliosis that attenuated within one week with no glial scar evident upon recovery from the damage, suggesting that astrogliosis differs in zebrafish when compared to its mammalian counterparts [169,170]. The lack of prolonged inflammation or persistence of a scar at the injury site should be considered for identifying specific mechanisms that promote the resolution of glial scar and inflammation. In addition, myelin molecules can inhibit axonal outgrowth and affect differentiation of oligodendrocyte precursor cells during remyelination [171]; therefore, while clearing of myelin debris is key to restoration of axonal outgrowth, this is not required in olfactory axon plasticity since these processes are not myelinated. 

Neuronal loss leads to a prolonged neuroinflammatory response in mice, characterized by activated microglia expressing CD68 and CD45 with elevated levels of inflammatory signals such as cytokines, chemokines, and complement proteins [166]. Debris clearance through phagocytosis is an essential function that is more complex than creating physical space for regeneration, but rather plays a role in the reorganization of neuronal circuits that trigger repair [147]. Microglial cells respond and migrate towards CX3CR1 in support of endangered neurons. With this, microglia responding to complement receptor C3 targeting unwanted synapses may play a role in synapse removal, as seen during development [172]. 

Many microglial studies in fish examine photoreceptor regeneration [112,158,173]. These types of studies on cellular and molecular mechanisms and inflammation have the potential to elucidate the regenerative potential of mammalian tissues [174,175]. In zebrafish, acute inflammation is necessary to induce neuronal regeneration [121,176]. In acute injury, microglia will migrate towards the lesion within a few hours. This migration is functionally significant as a loss in CXCR3 function in mice, microglia do not migrate and there is no loss of dendrites [177]. Degenerating axons are not removed autonomously but rather require a trigger signal to be removed by microglia. In an experiment where an axonal lesion was performed to facial nerve motoneurons in rats, there was a glial cell-mediated removal of synapses from the perikaryon and dendrites of affected cells [178,179]. 

Microglia rapidly respond to injury-dependent changes in neuronal activity, altering their morphology to activated, amoeboid, and become phagocytic to remove nonfunctional neuronal elements after injury during a pro-inflammatory response that may involve the complement system; microglia-specific receptor of chemokine fractalkine CXC3CL1, CX3CR1; TNF-α; IL-1β; and MHC-1 [54,180]. Repopulation and proliferation of microglia occur as they enter an anti-inflammatory phase and encourage neuronal outgrowth and regeneration through anti-inflammatory signaling. For example, purinergic signaling is important to target processes for removal, while ATP may act as a noninflammatory signal [54]. Anti-inflammatory signaling may include TGF-β, IL-10, and ATP when microglia return to predominantly a resting ramified morphology, where they continue to survey the environment [72]. 

Zebrafish are an emerging real-time model to study microglia and neurodegenerative disorders such as Parkinson’s disease and Alzheimer’s disease. These fish possess a high degree of neuroanatomical, neurochemical, and emotional/social behavioral resemblance with humans, serving as an ideal simulator for many pathologies as well as tauopathy [50,181,182,183]. Neurodegeneration induced by Amyloid-β results in activated microglia to prevent synaptic degeneration and promote neurogenesis, and the zebrafish brain possess molecular mechanisms that underlie a successful regeneration response after neurodegeneration [184]. 

Easy manipulation and visualization of optically translucent zebrafish embryos allow for real-time imaging of microglial activity. Markers for macrophages in live imaging zebrafish studies include transgene promoter mpeg1 and mfap4, similar to their mammalian counterpart studies [87,185]; however, microglial markers in zebrafish differ slightly from mammalian markers and include myeloid marker lymphocyte cytosolic plastin 1 (lcp1) and 4C4 [72,90,186]. It is important to note that the diversity of macrophages and microglial subtypes in zebrafish is an emerging area of study, and there is not unanimous agreement on reliable markers for each cell type. Throughout this review, we have cited sources that use primarily mpeg1 and mfap4 to label macrophages; lcp1 to label microglia, despite it being regarded as a pan-leukocyte marker; and 4C4 to label microglia exclusively in fish. Microglial-mediated neurodegeneration can be observed in the form of phagocytosis [50]. Studies of mitochondrial fragmentation and oxidation have been performed to examine the vulnerability of axonal degeneration through confocal imaging [187,188], and combined with time-lapse imaging, extrinsic cells have been revealed to play a role in the dynamics of the degeneration and regeneration of axons in the zebrafish larvae [189]. Real-time imaging using zebrafish has revealed many phenomena regarding plasticity and molecular mechanisms for possible avenues for treating degeneration.

As mentioned before, the timely response of innate immune cells can be a prerequisite for tissue regeneration, through removal of cellular debris and control of inflammation. Unlike mammals, zebrafish have a high regenerative capacity to restore tissue, notably brain and cardiac tissues [96,111,122]. Thus, identifying conditions and relative timing of innate immune cell response that promote the regeneration of zebrafish neuronal tissue may provide insight on understanding the lower regenerative potential in mammals. 

## 5. Conclusions

Recovery of neuronal function after brain disease and injury is one of the most common challenges in clinical settings. The innate immune cells of the peripheral nervous system share similarities with that of the CNS; therefore, it is imperative to understand the functions of both macrophages and microglia and study these two cell types collectively. Novel findings regarding the regenerative ability of fish in relation to microglial and macrophage activation could provide a handle for modifying the toxic and chronic neuroinflammatory processes that ensue after brain disease and injury in mammals. The studies reviewed here illuminate the microglial and macrophage characteristics after injury in various anatomical and functional architectures of the zebrafish model, contrasting with the lower regenerative potential found in mammalian models. Understanding how these innate immune cells function in a highly plastic model provides a foundation for future studies for understanding neuroinflammation in the context of recovery and regeneration after disease and injury. 

## Figures and Tables

**Table 1:** Zebrafish Regeneration Studies on Macrophage and Microglial Involvement.

Tissue	Life Stage	Cell Type	References
Heart	Adult	Macrophage	[91,92,93,94,95,96,97,98,99]
Fin	Larvae	Macrophage	[89,97,100,101],
Fin	Adult	Macrophage	[87,97,102,103]
Hair Cell	Larvae	Macrophage	[104,105,106,107,108]
Retina	Adult	Microglia	[109,110,111,112]
Spinal Cord	Larvae	Microglia	[90,113,114,115]
Spinal Cord	Adult	Microglia	[116,117,118,119]
Brain (telencephalon, olfactory bulb)	Adult	Microglia	[72,85,120,121,122,123,124,125]

Study of macrophages and microglia in relation to regeneration after injury is popular in both larvae and adult zebrafish models. There is noted difficulty in distinguishing microglia from infiltrating macrophages in many zebrafish studies, as semantic variability is prevalent throughout the literature.
